# Investigation of Ferromagnetic and Ferroelectric Properties in Binderless Cellulose/Ni Laminates for Magnetoelectric Applications

**DOI:** 10.3390/polym14245347

**Published:** 2022-12-07

**Authors:** Manseong Song, Su-Chul Yang

**Affiliations:** Department of Chemical Engineering (BK21 FOUR), Dong-A University, Busan 49315, Republic of Korea

**Keywords:** binderless, magnetoelectric, laminate, cellulose, nickel, ferromagnetic, ferroelectric

## Abstract

According to reported polymer-based magnetoelectric (ME) laminates, which generate voltage via an external magnetic field, a binder is indispensable for the adhesion between phases. However, if the binder is excluded, the ME response is expected to improve via efficient strain transfer from the magnetostrictive phase to the piezoelectric phase. Nevertheless, an understanding of the binderless state has not yet been addressed in polymer-based ME laminates. In this study, cellulose/Ni (CN) laminates were designed to obtain binderless polymer-based ME laminates. The surface properties of Ni foil desirable for the anchoring effect and the electrostatic interactions required for binderless states were determined via heat treatment of the Ni substrate. Moreover, to confirm the potential of the binderless laminate in ME applications, the ferromagnetic and ferroelectric properties of the CN laminates were recorded. Consequently, the CN laminates exhibited remnant and saturation magnetizations of 29.5 emu/g and 55.2 emu/g, respectively. Furthermore, the significantly increased remnant and saturation polarization of the CN laminates were determined to be 1.86 µC/cm^2^ and 0.378 µC/cm^2^, an increase of approximately 35-fold and 5.56-fold, respectively, compared with a neat cellulose film. The results indicate that multiferroic binderless CN laminates are excellent candidates for high-response ME applications.

## 1. Introduction

The magnetoelectric (ME) effect has attracted considerable attention from various research groups owing to its unique characteristics such as electrical polarization under an applied magnetic field. Diverse studies on ME materials have been reported such as energy-harvester-assisted magnetic fields, high-sensitivity magnetic sensors, wireless electronic devices, memory devices, transducers, actuator applications, and even active cancer-targeting/drug-delivery systems [1,2,3,4,5,6,7]. 

As a class of ME materials, an ME composite is defined as a complex containing piezoelectric (PE) materials that generate voltage under stress and magnetostrictive (MS) materials whose physical shape is distorted by an external magnetic field [8]. Numerous investigations on ME composites have been conducted owing to their relatively superior ME voltage at room temperature and economic feasibility compared to single-phase ME compounds [9].

Because of their relatively efficient strain transfer, low-temperature processing, easy fabrication, and excellent ME output voltages among the various structures of ME composites, (2-2) laminate structures, in particular, have been intensively explored by many groups [10].

The principle of the ME effect on an ME laminate is as follows: When the composite is exposed to an external bias magnetic field, the MS phase causes mechanical stress on the adjacent PE phase by deforming its shape. Thus, mechanical stress immediately leads to electrical polarization in the PE phase. Consequently, applying a magnetic field can control the generated bias voltage. Additionally, the degree of strain transfer must be considered because the ME effect occurs via elastic interaction at the interface of the phases [11].

(1) $$ME\;effect=\frac{strain\;change}{magnetic\;field}\times strain\;transfer\times \frac{electrical\;polarization}{strain\;change}$$

Regarding the (2-2) laminates, the PE phase of ME laminate composites can be prepared using ceramic or polymer materials. Although ceramic-based PE phases exhibit outstanding PE characteristics, researchers have recently utilized PE polymers such as cellulose, poly(vinylidene fluoride) (PVDF), poly(vinylidene fluoride-trifluoroethylene) (PVDF-TrFE), and poly(vinylidene fluoride-hexafluoropropylene) (PVDF-HFP) and its copolymers as PE materials owing to their low-temperature processing, simple fabrication, comparable low cost, and high flexibility leading to their high feasibility in multiple applications in the industrial field [12,13,14,15,16,17]. 

Recently, several studies using cellulose as a PE phase have been reported in the field of polymer-based ME composites because cellulose is regarded as an easily extractable natural polymer with inherent PE properties. Particularly, in the first attempt to utilize cellulose as a PE material for the ME composite, cellulose as a PE matrix demonstrated a stable film state of a 3-0-type cellulose-based ME composite, exhibiting a maximum magnetization value of 12.96 emu/g. For cellulose-based ME laminates, cellulose/epoxy/metglas laminates have been reported to generate excellent ME coefficients of approximately 1.5 V/(cm·Oe) in the resonance frequency mode. [14,18] In addition, according to the literature on the cooperation mechanism between polymers and other materials, the hydroxyl group on the surface of cellulose can conveniently form primary or secondary bonding with other components [19,20,21]. Furthermore, a remarkable feature of cellulose is that the cellulose does not require a post-poling process, which is regarded as an essential procedure for typical piezoelectric polymers to be polarized during fabrication. Therefore, the use of cellulose reduces the costs for fabrication of the composites at industrial sites [18,22,23]. 

Nonetheless, the reported polymer-based ME laminates have used epoxy-based binders to combine the PE and MS phases. In fact, the thinner the binder and the higher Young’s modulus is in the binder, the greater the ME effect that can be induced [24]. Therefore, excluding the binder at the interface of the ME laminate can be expected to positively affect the ME response owing to the fully transferred strain from the MS phase to the PE phase and the electrical interaction between the phases.

**Figure 1:**
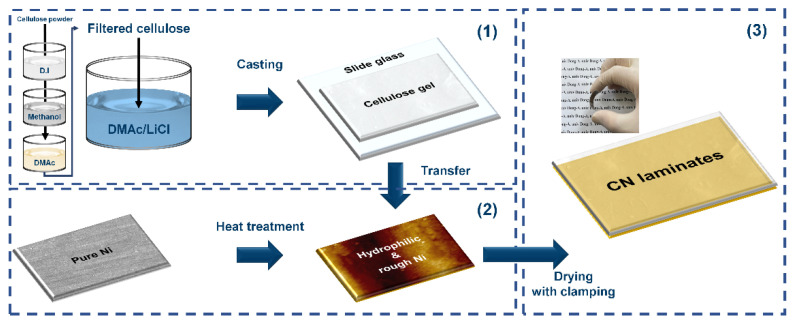
Schematics of the overall fabrication process of the CN laminates.

In this study, as a key to improving the strain transfer and encouraging electrical interaction at the interface, a cellulose/Ni (CN) laminate was designed without any adhesive, as shown in Figure 1. (1) Cellulose gel was prepared through consecutive processes consisting of the swelling sequence in a DMAc/LiCl solvent system after solvent exchange stages in several solvents due to the poor solubility of raw cellulose powder. Thereafter, the cellulose solution was casted on slide glass and pre-dried to obtain gel-type cellulose. (2) Ni was chosen for the MS phase because it exhibits magnetostrictive behavior even at a low magnetic field of less than 100 Oe [25,26]. Heat treatment of a Ni substrate was performed at different temperatures to regulate oxygen groups on the surface and the surface roughness. (3) The prepared cellulose gel and heat-treated Ni substrate were then completely clamped together and dried. Finally, the CN laminate was fabricated through a mild process without high pressure or high temperature, which could provoke undesirable damage on the samples, as shown in the inset displaying a real image of the flexible CN laminate in Figure 1. The hydrophilicity and topography of the heat-treated Ni substrates were investigated to determine the mechanism of the binderless state of the CN laminates. Moreover, their ferroelectric and ferromagnetic properties were explored to assess the possibility of using CN laminates for ME applications. 

## 2. Experimental Procedure

### 2.1. Materials

α-cellulose powder (powder size > 70 µm, Sigma-Aldrich, Saint Louis, MO, USA), anhydrous dimethylacetamide (DMAc; anhydrous 99.8%, Sigma-Aldrich, Saint Louis, MO, USA), lithium chloride (LiCl; ACS reagent ≥ 99.9%, Sigma-Aldrich, Saint Louis, MO, USA), methanol (anhydrous 99.8%, Sigma-Aldrich, Saint Louis, MO, USA), and Ni foil (Purity > 99.9%, MTI Korea Co., Seoul, Republic of Korea) were purchased.

### 2.2. Heat Treatment on Ni Surface

The heat treatment of Ni substrate was conducted via thermal oxidation in a furnace. Before the process, the Ni substrates were tailed to dimensions of 32 × 8 × 0.03 mm. The applied temperature was varied at 100 °C, 200 °C, 300 °C, 400 °C, and 500 °C for each sample and maintained for 1 h. Thereafter, the cooling sequence was performed via natural cooling to room temperature. In this study, the pure Ni and heat-treated Ni substrates oxidized at 100 °C, 200 °C, 300 °C, 400 °C, and 500 °C were named PN, Ni100, Ni200, Ni300, Ni400, and Ni500, respectively.

### 2.3. Preparation of Cellulose Gel

To achieve excellent solubility, the α-cellulose powder was pretreated via the solvent exchange method [27] prior to dissolving α-cellulose in the final solvent system. First, 0.5 g of α-cellulose powder was stirred in 100 mL of deionized water (D.I) for 1 h at 40 °C. The suspension was then filtered by solid suspension vacuum filtration using a 0.8 µm pore size cellulose acetate membrane. These processes were performed twice using the same solvent. Subsequently, the filtered powder was added to 100 mL of methanol and stirred for 45 min followed by filtration. Methanol exchange was conducted twice. The same procedure was performed twice with 100 mL of DMAc in each case. The final swelling process was continued overnight during the final solvent exchange procedure in DMAc. Subsequently, to obtain fully dissolved cellulose, the DMAc/dried-LiCl solvent system was prepared at a constant temperature of 40 °C. In the final solution stage, the added quantities of DMAc and LiCl were 4 and 45.5 g, respectively. The filtered final cellulose suspension was added to the prepared DMAc/dried-LiCl solvent system with constant stirring for 1 d at room temperature. The solution was cast on a glass slide and pre-dried for 24 h in a fume hood at room temperature. The pre-dried cellulose gel was rinsed during the cleaning process with an isopropyl alcohol (IPA)/D.I mixture (1:3 volume ratio). Finally, the washed cellulose gel was immersed in D.I for 3 d to remove salt. To eliminate the excess salt completely, D.I was replaced every 24 h during the immersion process. Finally, the cleaned cellulose gel was tailed to 40 mm × 10 mm using a bistoury. 

### 2.4. Fabrication of CN Laminate

The prepared cellulose gel and Ni substrate overlapped with strong clamping between the hydrophobic Teflon-adhesive-coated slide glasses. Conventional metal clips provided a strong clamping force. The samples were dried in an oven for 12 h while clamped. The neat cellulose (NC) film used for comparing the chemical and ferroelectric properties was fabricated via the same procedure as the CN laminates but without the Ni substrate. For the electrode processes, Al foil was attached to the surface of the cellulose film.

### 2.5. Characterizations

The morphologies of the surfaces and cross-sections of the NC, heat-treated Ni substrates, and CN laminates were investigated using field emission scanning electron microscopy (FE-SEM; Scios2, Thermo Fisher Scientific, Waltham, Middlesex County, MA, USA) at an accelerating voltage of 10 kV. The oxygen fractions on the surface of the PN and heat-treated Ni substrates and the elemental distributions on the cross-section of the CN laminates were observed using energy-dispersive X-ray spectroscopy (EDX; Scios2, Thermo Fisher Scientific, Waltham, Middlesex County, MA, USA) at an accelerating voltage of 15 kV. The 3D topography images of the PN and heat-treated Ni substrates were obtained using atomic force microscopy (AFM; Innova, Bruker Co., Billerica, Middlesex County, MA, USA). Scanning was conducted in the tapping mode with a scan size of approximately 5 × 5 μm^2^ and a scan rate of 10 μm/s. Water contact angle measurements (KrÜss DSA10, KRÜSS Scientific Instruments Inc., Hamburg, Germany) using a video camera and image analysis software (KRÜSS Scientific Instruments Inc., Hamburg, Germany) were conducted to evaluate the hydrophilicity of the PN and heat-treated Ni substrates. Water droplets (10 μL) were used for surface measurements. The X-ray diffraction (XRD) patterns of the PN and heat-treated Ni substrates were analyzed using an X-ray diffractometer (MiniFlex600, Rigaku, Tokyo, Japan) with CuKα (λ = 1.5406 Å) radiation. The Fourier transform–infrared (FT–IR) spectra of the NC and CN laminates were obtained using an FT–IR spectrometer (Nicolet iS 20, Thermo Fisher Scientific Inc., Waltham, MA, USA) in the attenuated total reflectance mode in the range of 600–4000 cm^−1^. 

### 2.6. Ferromagnetic and Ferroelectric Measurements

The magnetic hysteresis loops of the PN, heat-treated Ni substrates, and CN laminates were recorded using a vibrating sample magnetometer (VSM) (Model 7404, Lake Shore Cryotronics Inc., Westerville, OH, USA) at room temperature. Polarization–electric field (P–E) curves of the CN laminates were obtained using a ferroelectric tester (Precision LC II, Radiant Technology Inc., Albuquerque, NM, USA) in the range of ±20 kV/mm with a 10 ms hysteresis period.

## 3. Results and Discussion 

**Figure 2:**
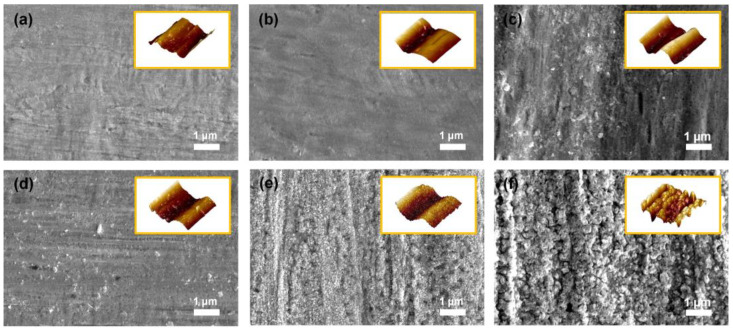
FE–SEM images of the top-view morphology of (**a**) PN, (**b**) Ni100, (**c**) Ni200, (**d**) Ni300, (**e**) Ni400, and (**f**) Ni500. The yellow boxed insets in each image indicate AFM 3D topography for each sample.

The surface roughness of the substrate is regarded as a crucial factor for the binderless state because binderless adhesion depends on the anchoring effect, in which the elastic polymer matrix is fixed within the rough structures on the metal surface by penetration [28]. Furthermore, the adhesion strength between the phases increased as the surface roughness increased, owing to the enlarged surface area for contact [29,30]. According to the literature, the degree of surface roughness of metal can be easily controlled by heat treatment owing to the indiscriminately organized surface metal oxide in proportion to the oxidation time and temperature [31,32,33]. Therefore, FE–SEM and AFM analyses were conducted to confirm the changes in the surface topography by the applied temperature. As shown in Figure 2a, the PN exhibited a convex morphology on the surface even before thermal oxidation. As seen in Figure 2b,c, no noticeable changes were detected until the applied temperature was 200 °C or less. However, after the heat treatment at temperatures over 300 °C, the surface of the Ni substrate became rougher with increasing the oxidation temperature. The small-sized cracks on the surface began to emerge when the applied oxidation temperature was above 300 °C. The nanoscale bump structure was detected in both the FE–SEM and AFM images. In particular, as shown in Figure 2d,e, the surfaces of Ni300 and Ni400 displayed both a bulk convex morphology and nano-bump structures in contrast to Ni500 (Figure 2f). The rough surface of the substrates with a structure containing bulk convex distortion and nano-bumps was the main factor for the anchoring effect.

**Figure 3:**
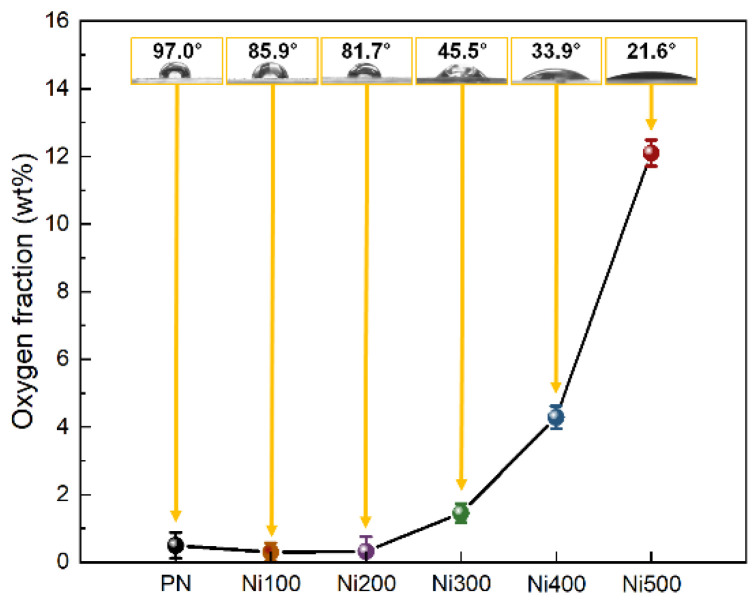
Oxygen weight fraction plots of PN, Ni100, Ni200, Ni300, Ni400, and Ni500 measured using EDS mapping analysis. The yellow boxed insets at each point in the images illustrate the water contact angle and real images of droplets on the surface for each sample.

In addition to physical morphologies, the chemical characteristics of the surface should also be considered when investigating the origin of binderless adhesion because the adhesion between the metal and polymer is caused by not only physical effects but also hydrogen bonds with electrostatic interactions [21]. In general, with increasing the oxidation temperature, the oxygen fraction on the Ni surface increased owing to oxygen atoms entering the Ni crystal lattice during the thermal oxidation. As shown in Figure 3, no noticeable change in the oxygen fraction was observed when the applied oxidation temperature was 200 °C or less; however, the water contact angle (WCA), which represents the surface hydrophilicity, gradually decreased as the oxidation temperature increased. For the WCA, this was because of the increased hydrophilicity of the substrates owing to the Wenzel state, which occurs when the gap on a surface is filled with water molecules via active electrostatic interactions if the substrate contains functional groups with a high surface energy [34]. Furthermore, the formed roughness (confirmed in Figure 2) provided an expanded active site, and abundant oxygen assisted in the surface interaction with water molecules. Above 300 °C, distinguishable oxygen fraction and WCA changes of approximately 1.46 wt% and 45.5° were observed. The oxygen fraction sharply increased, while the WCA decreased with increasing the temperature. This implied that a threshold point exists to obtain effective hydrophilicity, and binderless adhesion would not be realized until the Ni is heat-treated at more than 300 °C. In summary, these results indicated that the hydrophilic treatment was completed via facile thermal oxidation.

**Figure 4:**
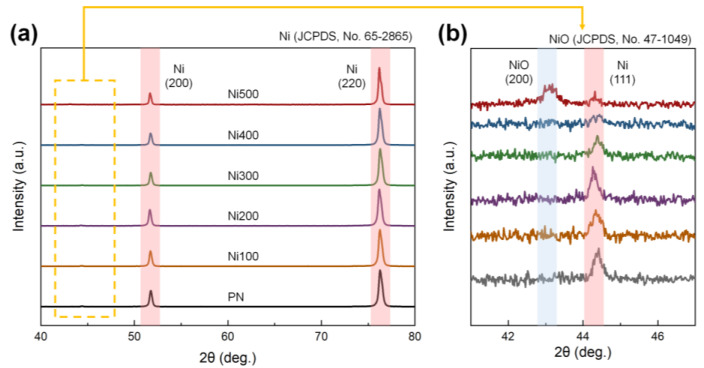
(**a**) XRD diffraction patterns of PN, Ni100, Ni200, Ni300, Ni400, and Ni500 in the range of 40–80°. (**b**) Magnification of the XRD diffraction patterns in the range of 41–47°. The red and blue lines in each figure indicate the leading peaks of Ni and NiO, respectively.

Although thermal treatment promotes the conditions required for binderless laminates, it may also result in improper crystallization. For instance, the formation of NiO, which exhibits antiferromagnetic features, could be facilitated during thermal oxidation, weakening the total ferromagnetic properties in the MS [35]. NiO also possesses a brittle mechanical character; thus, it can lead to inefficient strain transfer caused by defects and voids between the phases during mechanical deformation [36]. Because of the poor magnetic and mechanical properties reported, the rapid formation of NiO on the MS phases is inappropriate for the ME properties of laminates. Therefore, to confirm the presence of impurities on the surface of the substrate, XRD patterns of PN, Ni100, Ni200, Ni300, Ni400, and Ni500 were investigated, as shown in Figure 4a. In the XRD pattern, distinct peaks of (200) and (220) were observed at 51.74° and 76.26°, respectively, corresponding to Ni (JCPDS, No. 65-2865) [37]. To observe the details of the XRD patterns of Ni and NiO, a specific region of the XRD pattern was magnified. The XRD pattern in Figure 4b demonstrates that an additional peak that did not belong to pure Ni was observed only in the case of Ni500. The peak corresponding to the (200) plane of NiO appeared at 43.14°, which was in accordance with NiO (JCPDS, No. 47-1049). A peak of (111) at 44.42°, which was consistent with Ni, was also detected [38]. This diffraction peak implied that the crystallinity of NiO in the substrates was too low to be detected when the oxidation temperature was below 500 °C. Thus, most of the heat-treated Ni substrates did not exhibit clear NiO crystals, indicating only a higher number of oxygen groups and increased roughness, both of which are required for a binderless state.

**Figure 5:**
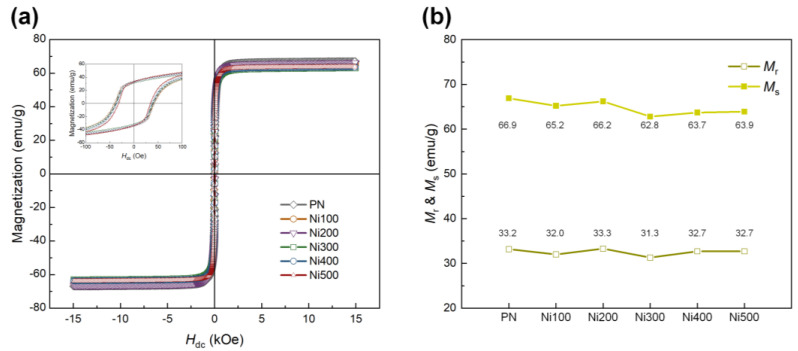
(**a**) M −H hysteresis loops of PN, Ni100, Ni200, Ni300, Ni400, and Ni500 in the range of ±15,000 Oe. The inset shows the small range of magnetization behavior for each sample according to the applied external magnetic field in the range of ±100 Oe. (**b**) Values of *M*_r_ and *M*_s_ for each sample.

Because the ferromagnetic properties of the Ni substrates used as the MS phase directly affect the ME properties, the magnetization behavior of MS materials according to the applied external magnetic field must be explored. M–H loops were recorded for all samples to investigate the ferromagnetic properties of the PN and heat-treated Ni substrates, as shown in Figure 5a. After thermal oxidation, the ferromagnetic properties of the heat-treated Ni substrates appeared to be slightly weaker than those of the PN. This was because of the formation of antiferromagnetic NiO, which led to a decrease in the amount of pure Ni consumed during oxidation [39]. Figure 5b shows slight differences in the ferromagnetic parameters, including remnant magnetization (*M*_r_) and saturation magnetization (*M*_s_), for all the samples. For all the heat-treated Ni substrates, the calculated maximum differences in *M*_r_ and *M*_s_ compared with the PN were −5.70% and −4.87%, respectively. Despite the slight reduction in *M*_r_ and *M*_s_ in the overall heat-treated Ni substrates, these still exhibited ferromagnetic behavior, while the degree of decrease was negligible.

**Figure 6:**
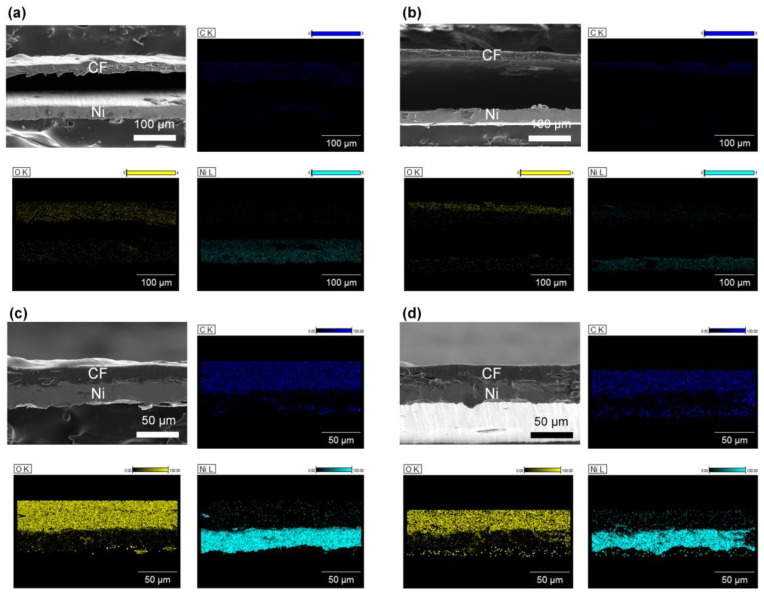
FE −SEM images of the cross-view morphology with EDS profiling of (**a**) CN200, (**b**) CN500, (**c**) CN300, and (**d**) CN400. EDS profiles are according to each FE −SEM image. The PE and MS phases were identified by detecting the distribution of carbon, oxygen, and nickel atoms.

Through the optimization of the hydrophilicity and surface roughness of the Ni substrate, it was concluded that Ni300 and Ni400 were the only samples that could achieve binderless adhesion. This might be because these substrates possessed hierarchically structured surfaces (which was verified by the AFM and FE–SEM images shown in Figure 2) with a sufficient oxygen fraction (which was confirmed by the EDX profiling in Figure 3). Therefore, the subsequent investigation was focused on CN300 and CN400, which contained Ni300 and Ni400 with cellulose film (CF), respectively. Prior to investigating the morphological features of the CN laminates, the surface morphology of the NC was examined to demonstrate whether the prepared CF consisting of CN was well prepared. As shown in Figure S1a, in the top-view image of NC, cracks that resulted in reduced polarization when external stress was applied were not observed on the surface. Furthermore, Figure S1b shows the stacked structures of cellulose that gave rise to the PE properties [40]. To verify the binderless state in the CN laminates, the overlapped laminates, including CN200 and CN500, containing Ni200 and Ni500 with CF, respectively, were also prepared. To capture the cross-sectional images, the MS and PE phases in the overlapped laminates were fixed using polyimide tape. This was because Ni200 and Ni500 could not achieve the binderless state as they did not fulfill the oxygen fraction and appropriate roughness requirements. Figure 6a–d display the cross-sectional images of each laminate, illustrating the obvious difference between the binderless and overlapped laminates. Despite the strong fixing provided by tape, the cross-sectional images of CN200 and CN500 show a colossal gap of more than 100 μm between the PE and MS phases, making strain transfer impossible [41]. In contrast, the cross-sectional images of CN300 and CN400 demonstrate that binderless adhesion was achieved, showing no gap between the phases. This binderless adhesion could be attributed to the physical anchoring effect brought about by the sufficient roughness with hydrogen bonds that originated from the appropriate oxygen fraction on the surfaces of Ni300 and Ni400.

**Figure 7:**
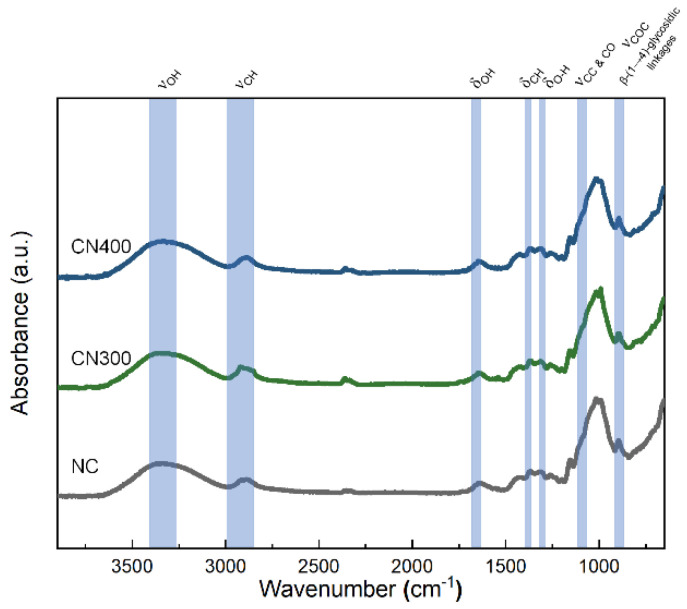
FT −IR spectra of NC, CN300, and CN400 in the range of 650–4000 cm^−1^.

Prior to reviewing the multiferroic properties of the CN laminates, the chemical structure of the PE phase should be revealed for the verification of non-impurities and appropriate preparation of the CF. In addition, the confirmation of emerged hydrogen bonding, which could be formed by oxygen atoms on the heat-treated Ni substates, should be conducted. Therefore, FT–IR analysis was performed to determine the surface chemistry of NC, CN300, and CN400. According to Figure 7, the investigated spectrum matched that of cellulose fibers reported in the literature [42]. The absorbance peak at 3337 cm^−1^ was because of the stretching of OH groups engaged in H-bonding in the cellulose molecules as well as interfacial hydrogen bonds with substrate surface. The absorbance peak at 2890 cm^−1^ corresponded to C–H stretching. The band at 1633 cm^−1^ was attributed to the OH bending of the absorbed water. In the low wavelength range, the CH_2_ and O–H bending peaks ware located at 1415 cm^−1^ and 1369 cm^−1^, respectively. The band at 1319 cm^−1^ was ascribed to C–C and C–O skeletal vibrations. Representative peaks of cellulose, indicating C–O–C stretching at the b-(1→4)-glycosidic linkages, were also observed at 1156 cm^−1^ and 896 cm^−1^. According to the observed intensity of the absorbance peak at 3337 cm^−1^_,_ noticeable differences among the samples were not observed because of the cellulose film, which had a too thick thickness for IR light to penetrate through and reach the interface. Nonetheless, the FT–IR spectra confirmed that the CN laminates were fabricated without any chemical impurities that could cause a functional decline of the PE phase. 

**Figure 8:**
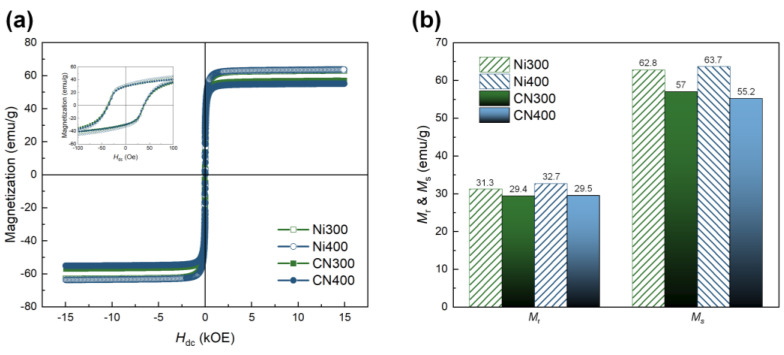
(**a**) M −H hysteresis loops of Ni300, Ni400, CN300, and CN400 in the range of ±15,000 Oe. The inset shows a small range of magnetization behavior according to the applied external magnetic field in the range of ±100 Oe. (**b**) Comparison of *M*_S_ and *M*_r_ between the heat-treated Ni substrates and CN laminates.

To identify the ferromagnetic properties, including *M*_r_ and *M*_s_, of the CN laminates, which are directly associated with the ME properties, M–H hysteresis loops of CN300 and CN400 were recorded in the range of ±15,000 Oe. They were compared with those of Ni300 and Ni400, as shown in Figure 8a. The *M*_r_ and *M*_s_ values for all samples are depicted in Figure 8b. The overall *M*_r_ and *M_s_* of CN laminates presented relatively diminished magnetic parameters because the mass of the laminates included that of the non-magnetic polymer matrix. Nevertheless, both the CN laminates exhibited conventional ferromagnetic behavior, displaying clear hysteresis loops and maintaining *M*_r_ and *M_s_*. Particularly, the *M*_r_ and *M*_s_ of CN300 and CN400 were higher than 29.4 emu/g and 55.2 emu/g_,_ respectively.

**Figure 9:**
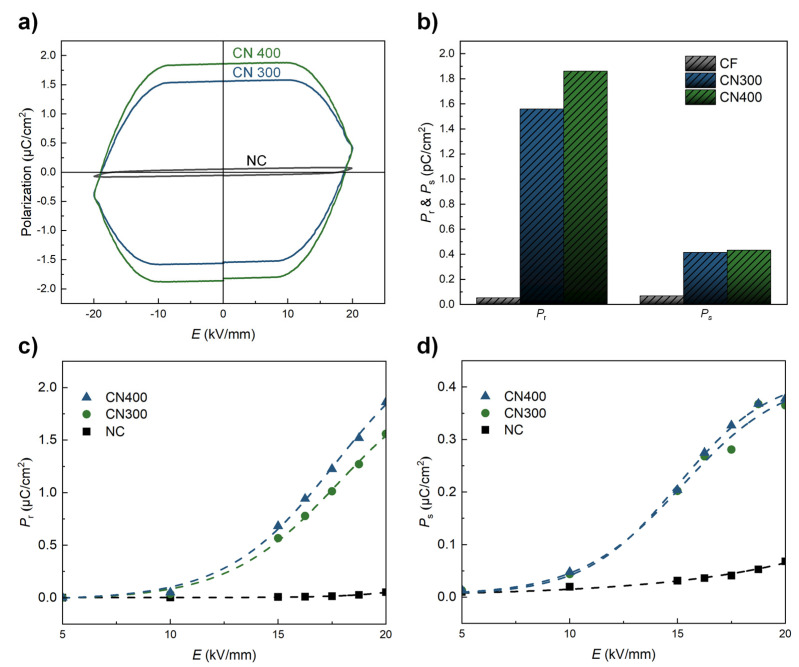
(**a**) P −E curves of NC, CN300, and CN400 in the range of ±20 kV/mm. (**b**) *P*_r_ and *P*_s_ plots of NC, CN300, and CN400 under a maximum electric field of 20 kV/mm. (**c**,**d**) *P*_r_ and *P*_s_ of each sample as a function of the applied electric field.

Finally, the ferroelectric properties (remnant polarization (*P*_r_) and saturation polarization (*P*_s_)) of NC, CN300, and CN400 were determined using the P–E hysteresis loops under an applied electric field of ±20 kV/mm, as shown in Figure 9. Before being attached to the heat-treated Ni substrate, the NC exhibited a relatively low ferroelectricity, exhibiting a typical P–E curve of cellulose-based PE materials, as shown in Figure 9a [43]. However, the ferroelectric properties of the CN laminates were significantly enhanced throughout the electric field range. The maximum ferroelectric properties measured under an external electric field of 20 kV/mm for each sample are illustrated in Figure 9b. The highest *P*_r_ and *P*_s_ values were approximately 1.86 µC/cm^2^ and 0.38 µC/cm^2^, respectively, for CN400. In addition, the values of *P*_r_ and *P*_s_ for CN400 corresponded to an increase of 35-fold and 5.56-fold, respectively, compared with those of the NC. This observation could be related to an electrical interaction between the phases caused by the absence of a binder. The improved ferroelectric properties of CN300 and CN400 could be attributed to the formation of additional hydrogen bonds, which promote an additional charge on the surface. [44] As shown in Figure 9c,d, both the increasing tendencies in the *P*_r_ and *P*_s_ plots indicated that the ferroelectric properties of CN400 were higher than those of CN300 over the entire range of the electric field. According to the EDS analysis, this may be attributed to the surface of Ni400 with a higher oxygen fraction, which could form more hydrogen bonds than Ni300. Therefore, the interaction based on binderless adhesion improved the ferroelectricity without any additional poling process. In addition, as hydrogen bonds connect the metal oxide and polymer, the reported length of the hydrogen bonds composed of oxygen and hydrogen atoms became less than 3 Å [21]. In other words, the adhesion between the phases was firmly maintained within an extremely close distance without detachment during the ferroelectric measurement. In addition, because the attached heat-treated Ni substrates acted not only as MS materials but also as electrodes in this study, the steady improvement in the ferroelectric properties with the electric field strength implied that the adhesion between phases was perfectly accomplished.

## 4. Conclusions

In this study, CN laminates were designed as a new approach to exclude binders in polymer-based ME laminates. The absence of the binder could promote an improved ME response owing to the fully transferred strain and electrical interaction between the phases. The Ni surface was modified via facile heat treatment to implement a binderless state of ME laminates, which could alter the surface properties including hydrophilicity and roughness. Therefore, in this study, the appropriate roughness and hydrophilicity of the heat-treated Ni substrates for binderless adhesion were ensured without significantly compromising the magnetic properties. Furthermore, the binderless state of the CN laminates was verified by cross-sectional images. Considering the ferromagnetic properties of the CN laminates, they exhibited ferromagnetic behavior, retaining their *M*_r_ and *M*_s_ values. In addition, it is noteworthy that the ferroelectric properties of the CN laminates were significantly improved compared to the NC laminates. This demonstrated that a relatively high ferroelectricity could be obtained owing to the additional hydrogen bonds between the phases induced by the oxygen groups on the surface of the heat-treated Ni substrates. Overall, the results concluded that not only the binderless state in ME laminates can be accomplished using a facile thermal treatment but also that the CN laminate is a promising candidate for various ME applications with multiferroic properties.

## Data Availability

Data are contained within this article.

## Supplementary Materials

The following supporting information can be downloaded at: https://www.mdpi.com/article/10.3390/polym14245347/s1, Figure S1: FE-SEM images in top-view and cross-view morphology of NC.
