# Supplementary material for: Investigation of Ferromagnetic and Ferroelectric Properties in Binderless Cellulose/Ni Laminates for Magnetoelectric Applications
Source: Polymers (Basel). 2022 Dec 7;14(24):5347. doi: 10.3390/polym14245347 (PMC9784961; doi:10.3390/polym14245347)
Supplement: Supplementary file 1 [file polymers-14-05347-s001.zip › polymers-2082386-supplementary.pdf]

Supporting Information for

Investigation of Ferromagnetic and Ferroelectric Properties in Binderless Cellulose/Ni Laminates for Magnetoelectric Applications

Manseong Song and Su-Chul Yang*

Department of Chemical Engineering (BK21 FOUR), Dong-A University, Busan, 49315, South Korea

* Correspondence: scyang@dau.ac.kr

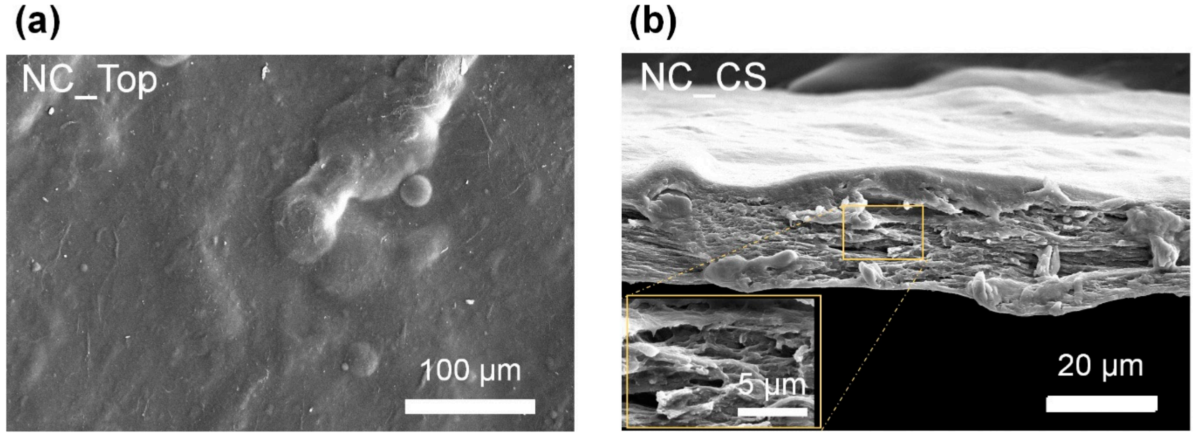

Figure S1. FE-SEM images of (a) top-view and (b) cross-view morphology of NC. The inset shows magnification image of stacked structure of NC.
